# Lack of adiponectin and adiponectin receptor 1 contributes to benign prostatic hyperplasia

**DOI:** 10.18632/oncotarget.19877

**Published:** 2017-08-03

**Authors:** Shi Fu, Huan Xu, Meng Gu, Chong Liu, Xiang Wan, Yanbo Chen, Qi Chen, Juan Zhou, Zhong Wang

**Affiliations:** ^1^ Department of Urology, Shanghai Ninth People’s Hospital, Shanghai Jiao Tong University School of Medicine (SJTUSM), Shanghai 200011, China

**Keywords:** BPH, adiponectin, obesity, AdipoR1, p90RSK

## Abstract

**PURPOSE:**

The incidence of benign prostatic hyperplasia increases among obese individuals, but few studies have fully explained the underlying mechanisms. Adiponectin has drawn much attention in recent years due to its protective role in obesity-related diseases. Here we aimed to investigate the possible molecular mechanisms and clinical significance of adiponectin in relation to benign prostatic hyperplasia.

**METHODS:**

We analyzed data from 98 Chinese men, including 48 BPH cases and 50 controls in a case-control study. Then, we utilized a tissue microarray analysis to examine expression of AdipoR1 and p-p90RSK in normal and hyperplastic prostate tissues. These studies were followed by various *in vitro* approaches to examine the anti-proliferation effect and signaling pathways of adiponectin involved in benign prostatic hyperplasia.

**RESULTS:**

Lower serum adiponectin levels were independently associated with larger prostate volume and an increased risk of benign prostatic hyperplasia. Benign prostatic hyperplasia tissues had a decreased expression of AdipoR1 and increased expression of p-p90RSK compared with normal prostate tissues. *in vitro*, adiponectin inhibited the proliferation of prostatic epithelial and stromal cells and arrested cells in the G_0_/G_1_ phase by decreasing phosphorylation of the MEK-ERK-p90RSK axis.

**CONCLUSIONS:**

Our results suggest a possible negative regulatory mechanism in which adiponectin signaling antagonizes ERK-mediated cell proliferation, and a deficiency in adiponectin could facilitate the proliferation of prostate cells and consequently contribute to benign prostatic hyperplasia.

## INTRODUCTION

Benign prostatic hyperplasia (BPH) is recognized as the most common pathologic condition in aging men and primarily leads to lower urinary tract symptoms (LUTS). Despite considerable research, the pathogenesis of BPH is multifactorial and largely unresolved. Histopathologically, BPH is considered to be a result of an imbalance of cell growth and death, which is mainly derived from hormone alterations, local inflammation and other pathological conditions [1, 2]. Increasing evidence suggests that obesity plays a key role in the development of BPH [3, 4], but the relationships between these two conditions are still poorly understood.

Adiponectin, which has been shown to have anti-proliferation, insulin-sensitizing, anti-tumor and anti-inflammation properties, is a kind of adipocytokines derived from adipose tissue [5]. Obese individuals have reduced adiponectin levels, and weight reduction increases adiponectin levels in circulation [6, 7]. AdipoR1 and AdipoR2 serve as two receptors for adiponectin that mediate glucose regulation, lipid metabolism, cell proliferation and apoptosis by activating the 5’ adenosine monophosphate-activated protein kinase (AMPK), peroxisome proliferator-activated receptor α (PPARα) and p38 mitogen-activated protein kinase (p38-MAPK) signaling pathways [8]. Numerous studies have found that people with obesity-related diseases have decreased expression of Adiponectin receptors in the lesion tissues [9]. Several studies have shown that adiponectin receptors are expressed in prostate cancer tissues and cell lines [10, 11]. This suggests an underlying role of adiponectin in the physiological or pathological processes of prostate diseases.

Consistent with previous evidence, we hypothesized that adiponectin deficiency is associated with the development of BPH. With this aim, we performed a case-control study to find links between adiponectin and BPH. We also compared the expression of AdipoR1 in normal tissues and hyperplastic tissues. Furthermore, we preliminarily investigated the anti-proliferation actions and mechanisms of adiponectin in prostate cell lines.

## RESULTS

### Lower serum adiponectin levels are associated with increased risk of BPH and prostate volume in a case-control study

**Figure 1:**
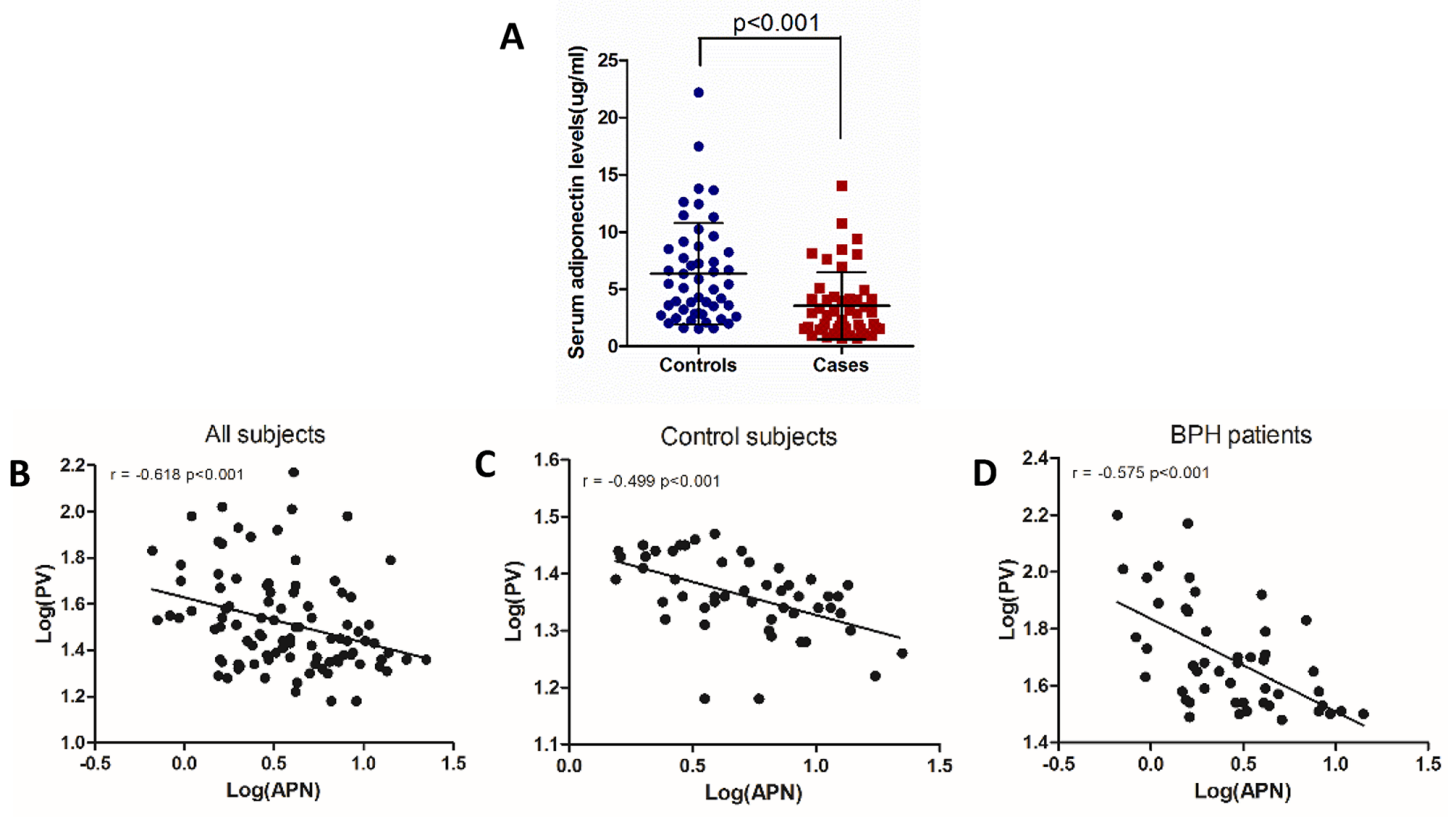
Serum adiponectin levels are negatively correlated with prostate volume (**A**) Significantly lower serum adiponectin levels were observed in BPH cases (n=48) compared with control subjects (n=50) (Mann-Whitney U test, p<0.001). (**B, C, D**) Correlations between serum adiponectin levels and prostatic volume among all subjects (r=-0.618, p<0.001), control subjects (r=-0.499, p<0.001) or BPH patients (r=-0.575, p<0.001). Two variables were log-transformed in the analysis (Pearson’s correlation coefficient). PV, prostate volume; APN, adiponectin.

A total of 98 patients were included in this hospital-based case control study as described in the Methods section. The epidemiological, clinical and analytical characteristics of the patients are shown in Table 1. Men with BPH tended to be older (p=0.001), heavier (p=0.003) and had less physical activity (p=0.024) than control subjects. Significantly decreased serum adiponectin levels (Figure 1A, p<0.001) and increased body mass index (BMI) (p=0.002) were observed in BPH cases compared with control subjects. Subjects with BPH deservedly had higher total prostate volume (TPV), prostatic specific antigen (PSA), the International Prostate Symptom Score (IPSS) and lower maximum urine flow rate (Q_max_) compared with the controls. The two groups were similar in terms of height, blood pressure, fasting blood glucose, cholesterol, triglycerides and high density lipoprotein (HDL), despite a slightly differing low density lipoprotein (LDL) levels.

**Table 1:** Descriptive characteristics of case and control subjects

Variables	Controls (n=50)	BPH (n=48)	P value
	Median (95% CI)	Median (95% CI)	
Continuous variables			
Age(years)	65.00(64.98-67.22)	69.00(67.97-70.86)	0.001*^b^
Height(cm)	1.70(1.70-1.73)	1.70(1.69-1.72)	0.923^b^
Weight(kg)	68.50(66.31-71.37)	75.00(71.71-76.79)	0.003*^a^
BMI(kg/m^2^)	23.60(22.57-24.39)	25.34(24.59-26.43)	0.002*^a^
FPG(mmol/L)	5.25(5.05-5.37)	5.05(4.96-5.25)	0.321^a^
SBP(mmHg)	124.50(120.70-126.87)	123.00(119.45-126.05)	0.581^b^
DBP(mmHg)	78.50(75.50-79.86)	76.50(73.51-78.37)	0.307^b^
PSA(ng/ml)	0.67(0.67-0.76)	2.83(2.77-4.30)	<0.001*^b^
Total cholesterol(mmol/L)	3.92(3.80-4.24)	4.38(4.02-4.61)	0.11^a^
Triglycerides(mmol/L)	1.21(1.24-1.66)	1.26(1.20-1.63)	0.831^b^
LDL(mmol/L)	2.67(2.45-2.81)	2.88(2.70-3.11)	0.041*^a^
HDL(mmol/L)	1.07(1.01-1.17)	1.08(1.01-1.21)	0.98^b^
FFA(mmol/L)	0.40(0.40-0.51)	0.38(0.33-0.41)	0.047*^b^
CRP(mmol/L)	3.75(3.60-4.77)	4.30(4.05-5.11)	0.188^b^
Adiponectin(μg/ml)	5.26(5.09-7.61)	2.78(2.69-4.40)	<0.001*^b^
Prostate volume(ml)	23.05(22.31-24.29)	45.04(46.66-63.61)	<0.001*^b^
Q_max_(ml/s)	25.65(24.97-27.74)	8.20(7.66-8.97)	<0.001*^a^
	Number (%)	Number (%)	
Categorical/Ordinal variables			
BMI(kg/m^2^) †			0.009*^c^
Underweight	3(6)	1(2.1)	
Normal	19(38)	6(12.5)	
Overweight	13(26)	13(27.1)	
Obesity	15(30)	28(58.3)	
Adiponectin quartiles^#^			<0.001*^c^
Q1≤1.972	3(6)	21(43.8)	
1.97<Q2≤3.727	15(30)	10(20.8)	
3.727<Q3≤7.104	15(30)	10(20.8)	
Q4>7.104	17(34)	7(14.6)	
IPSS			<0.001*^c^
None or light(0-7)	50(100)	0(0)	
Moderate(8-19)	0(0)	21(43.8)	
Severe(20-35)	0(0)	27(56.3)	
Physical activity			0.024*^c^
Sedentary	10(20)	21(43.8)	
Light	23(46)	19(39.6)	
Moderate	17(34)	8(16.7)	
Active	0(0)	0(0)	
Smoking status			0.867^c^
Current	20(40)	20(41.7)	
Former/Never	30(60)	28(58.3)	
Alcohol using status			0.715^c^
Current	18(36)	19(39.6)	
Former/Never	32(64)	29(60.4)	

^a^ Student’s T-test

^b^ Mann-Whitney U-test

^c^ χ^2^ test

† According to guidelines for the Asian Pacific population (International Association for the Study of Obesity, World Health Organization). Underweight, <18.5; normal, 18.5 to <23; at risk of obesity or overweight, 23 to<25; obesity, ≥25.

^#^Serum adiponectin levels were categorized in quartiles of its distribution.

*The significant parameters.

BPH, benign prostatic hyperplasia; BMI, body mass index; FPG, fasting plasma glucose; SBP, systolic blood pressure; DBP, diastolic blood pressure; PSA, prostatic specific antigen; LDL, low-density lipoprotein; HDL, high-density lipoprotein; CRP, C-reactive protein; Q_max_, maximum flow rate; FFA, free fatty acid.

We further performed bivariate linear correlations analysis determined by Pearson’s correlation coefficient. Among control subjects, TPV was negatively correlated with serum adiponectin levels (r=-0.499, p<0.01), and was positively correlated with BMI (r=0.323, p=0.022). Among BPH patients, correlative factors were similarly observed with respect to TPV (adiponectin, r=-0.575, p<0.01; BMI, r=0.297, p=0.04). However, adiponectin was not significantly correlated with Q_max_ and IPSS in control subjects or BPH patients. As expected, serum adiponectin levels were negatively correlated with BMI (r=-0.592, p<0.001). Figure 1B-1D show correlations between serum adiponectin levels and TPV in all subjects (r=-0.618, p<0.001), control subjects (r=-0.499, p<0.001) and BPH patients (r=-0.575, p<0.001).

Multivariate linear regression models as shown in Table 2 were conducted to estimate the predictors of TPV. Whether in a univariate model or adjusted multivariate model, adiponectin remained a significant negative predictor for TPV. Table 3 presents the results of univariate and multivariable logistic regression models. A significantly negative association of lower serum adiponectin levels with an increased incidence of BPH was shown in all models. The odds ratio of adiponectin was 0.795 (95%CI, 0.691 to 0.915) in univariate analysis. Multivariate adjustment for age, BMI, total cholesterol, triglycerides, LDL, HDL, C-reactive protein (CRP), smoking status, physical activity and alcohol usage strengthened the reduction in risk of BPH (OR, 0.671; 95%CI, 0.522 to 0.865; p<0.01). These findings suggest that lower serum adiponectin levels are associated with an increased risk of BPH and serum adiponectin levels are negatively associated with prostate volume rather than symptomatic BPH.

**Table 2:** Multivariate linear regression for predictors of prostate volume

Variables	Controls				Cases				All subjects			
	β	t	p	R^2^	β	t	p	R^2^	β	t	p	R^2^
Model 1												
Age*	0.094	0.293	0.771	0.454	0.814	1.097	0.279	0.444	2.182	4.190	<0.001	0.499
BMI	0.001	0.391	0.698		0.006	0.656	0.516		0.004	0.604	0.547	
TC	-0.041	-2.8887	0.006		-0.043	-1.232	0.225		-0.006	-0.240	0.811	
Triglycerides*	0.130	2.356	0.023		0.347	2.480	0.018		0.116	1.115	0.268	
LDL	0.002	0.116	0.908		-0.008	-0.201	0.842		0.001	0.041	0.968	
HDL*	-0.192	-1.802	0.079		0.298	1.352	0.184		0.135	0.811	0.420	
CRP	-0.002	-0.573	0.570		0.011	0.823	0.416		0.006	0.649	0.518	
Adiponectin*	-0.077	-2.028	0.049		-0.276	-3.299	0.002		-0.367	-5.941	<0.001	
Model 2 (stepwise method)												
Age*				0.249				0.331	2.167	4.388	<0.001	0.486
Adiponectin*	-0.115	-3.992	<0.001		-0.326	-4.772	<0.001		-0.395	-8.414	<0.001	

* Variables were log-transformed in the analysis.

β, regression coefficient; t, t-test for β; p, p values for t-test; R^2^, coefficient of determination.

BMI, body mass index; LDL, low-density lipoprotein; HDL, high-density lipoprotein; CRP, C-reactive protein; TC, Total cholesterol.

**Table 3:** Univariate and multivariate logistic regression for the risk of BPH in relation to serum adiponectin levels

Model	OR	95%CI	P value
1. Adiponectin, univariate	0.795	0.691 to 0.915	0.001
2. Adiponectin, BMI adjusted	0.836	0.713 to 0.982	0.029
3. Adiponectin, weight adjusted	0.832	0.712 to 0.973	0.021
4. Adiponectin, age adjusted	0.728	0.617 to 0.860	<0.001
5. Adiponectin, PA adjusted	0.809	0.679 to 0.964	0.018
6. Adiponectin, multivariate* adjusted	0.671	0.522 to 0.865	0.002

*Multivariate model included age, BMI, total cholesterol, triglycerides, LDL, HDL, CRP, smoking status, physical activity, alcohol using status.

BPH, benign prostatic hyperplasia; OR, odds ratio; CI, credibility interval; BMI, body mass index; PA, physical activity.

OR per one unit increase in each covariate.

### Lower AdipoR1 and higher p-p90RSK expression are associated with BPH

**Figure 2:**
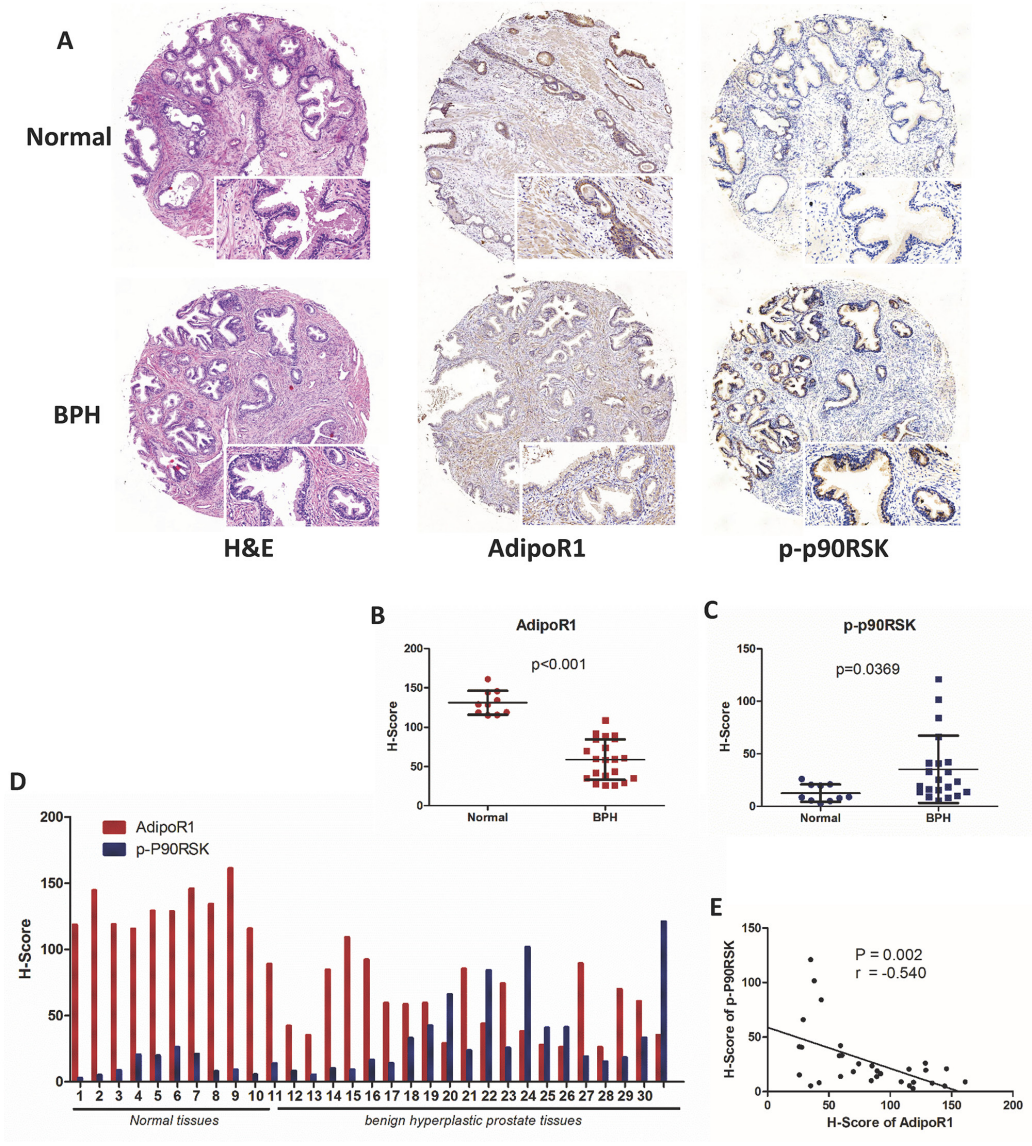
Expression of AdipoR1 and phospho-p90RSK in normal and BPH tissues (**A**) Analysis of tissue microarray (Biomax Inc, PR807b, containing 10 normal prostate tissues and 21 BPH tissues). H&E staining and IHC staining for AdipoR1 and phospho-p90RSK. Scale bars, 100 μm and 200 μm. (**B, C**) The relative expression of AdipoR1 (p<0.001) and phospho-p90RSK (p=0.0369) between normal and BPH tissues (Mann-Whitney test). IHC staining was semi-quantitated by H-Score as described in the Methods section. (**D)** **Expression of AdipoR1 and phospho-p90RSK on each sequential core.** The results are presented as the H-Score. (**E**) Linear correlation analysis of AdipoR1 and phospho-p90RSK expression (n=31; Pearson’s correlation coefficient, r=-0.540; p=0.002).

To test our hypothesis that adiponectin deficiency assists in the development of BPH, we performed a tissue microarray analysis using a human prostate tissue microarray containing normal (n=10) and benign hyperplastic prostate tissues (n=21). Immunohistochemical (IHC) staining showed that normal prostatic tissues had abundant expressions of AdipoR1 both in the prostatic epithelium and stroma but had less expression of phospho-p90RSK (a downstream target of ERK signaling) (Figure 2A). BPH tissues had decreased levels of AdipoR1 (p<0.001) and overexpression of phospho-p90RSK (p=0.0369) compared with normal tissues (Figure 2A-2D). A statistically significant negative correlation was seen between the H-Score values of AdipoR1 and phospho-p90RSK (r=-0.540, p=0.002, Figure 2E). Together, evidence form these studies reveals that adiponectin deficiency including a decrease of serum adiponectin levels and downregulation of AdipoR1, is associated with BPH.

### Adiponectin inhibits growth factor-mediated proliferation of prostatic epithelial and stromal cells *in vitro*

As described in the Methods section, we used two normal prostatic cell lines to investigate the effects of adiponectin and its receptors *in vitro*. Because PEpiCGS (a supplement of RWPE1 medium) and fetal bovine serum (FBS) contain different kinds of growth factors, we used 10ng/ml of human EGF instead of PEpiCGS and FBS to reduce the confounding factors in the following experiments.

**Figure 3:**
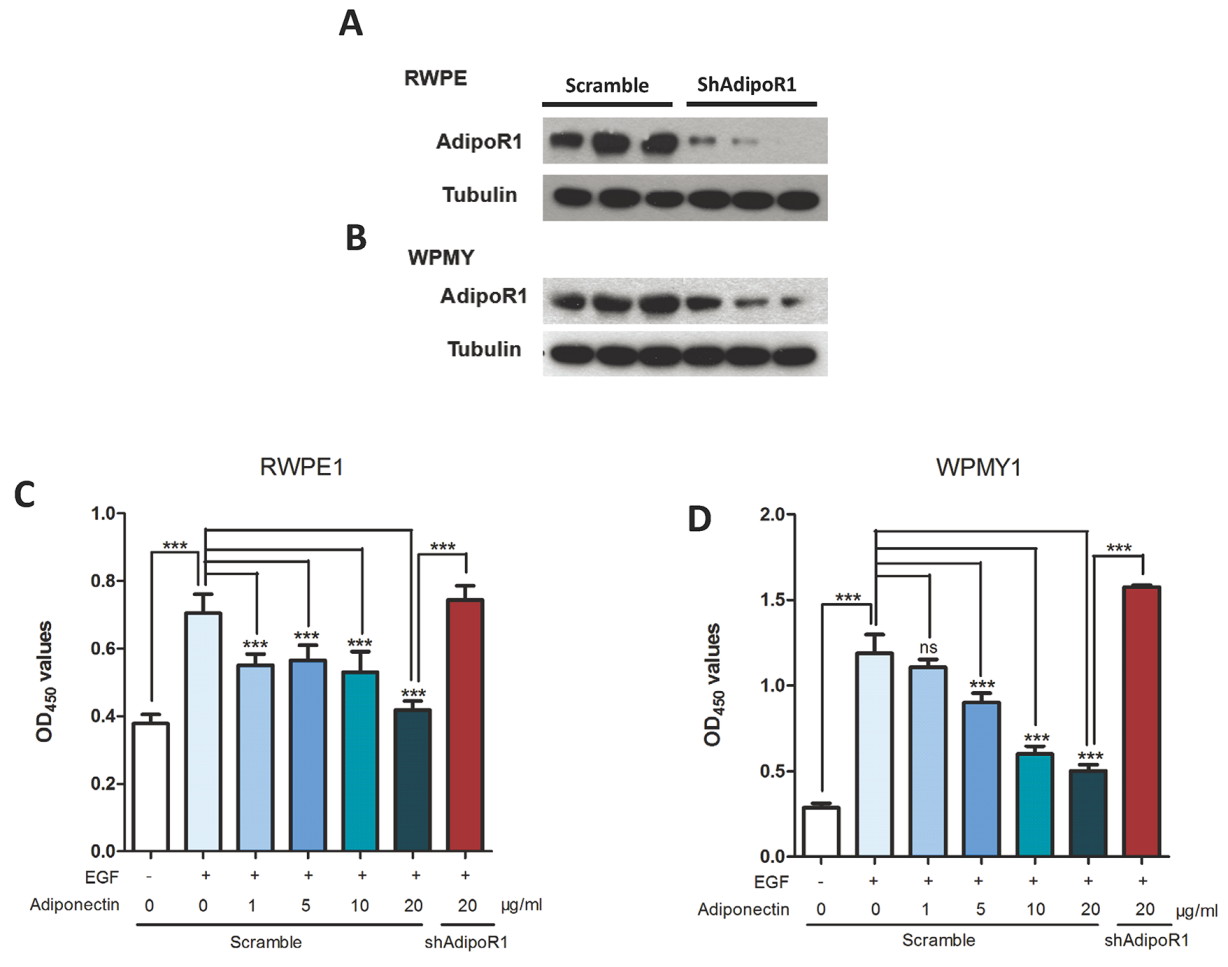
Adiponectin inhibits proliferation of prostatic epithelial and stromal cells (**A, B**) AdipoR1 knockdown efficiency was confirmed using western blotting. (**C, D**) CCK-8 proliferation analysis of RWPE1 and WPMY1 cells that were transfected with lentiviral vectors containing AdipoR1-shRNA or a scrambled sequence as a control. Cells were cultured in basic medium with or without 10ng/ml of EGF and then treated with 0, 1, 5, 10 or 20 μg/ml human recombinant adiponectin. The results are expressed as the mean±s.d. of three independent experiments (one-way analysis of variance followed by Dunnett post-tests; ***p<0.001, ns, not significant).

As shown in Figure 3C and 3D, proliferation of RWPE1 and WPMY1 was suppressed with an increase in adiponectin concentrations. In the absence of EGF, we found an attenuated anti-proliferation effect of adiponectin that might be due to the low multiplying rate of cells in the basal culture conditions. Moreover, knockdown of AdipoR1 led to recurrence of the EGF-induced proliferative effect. These results indicated that adiponectin has an anti-proliferation effect that is opposite to growth factors, and cells with adiponectin deficiency are more susceptible to growth-promoting factors.

### Adiponectin blocks the cell-cycle progression of prostatic epithelial and stromal cells

**Figure 4:**
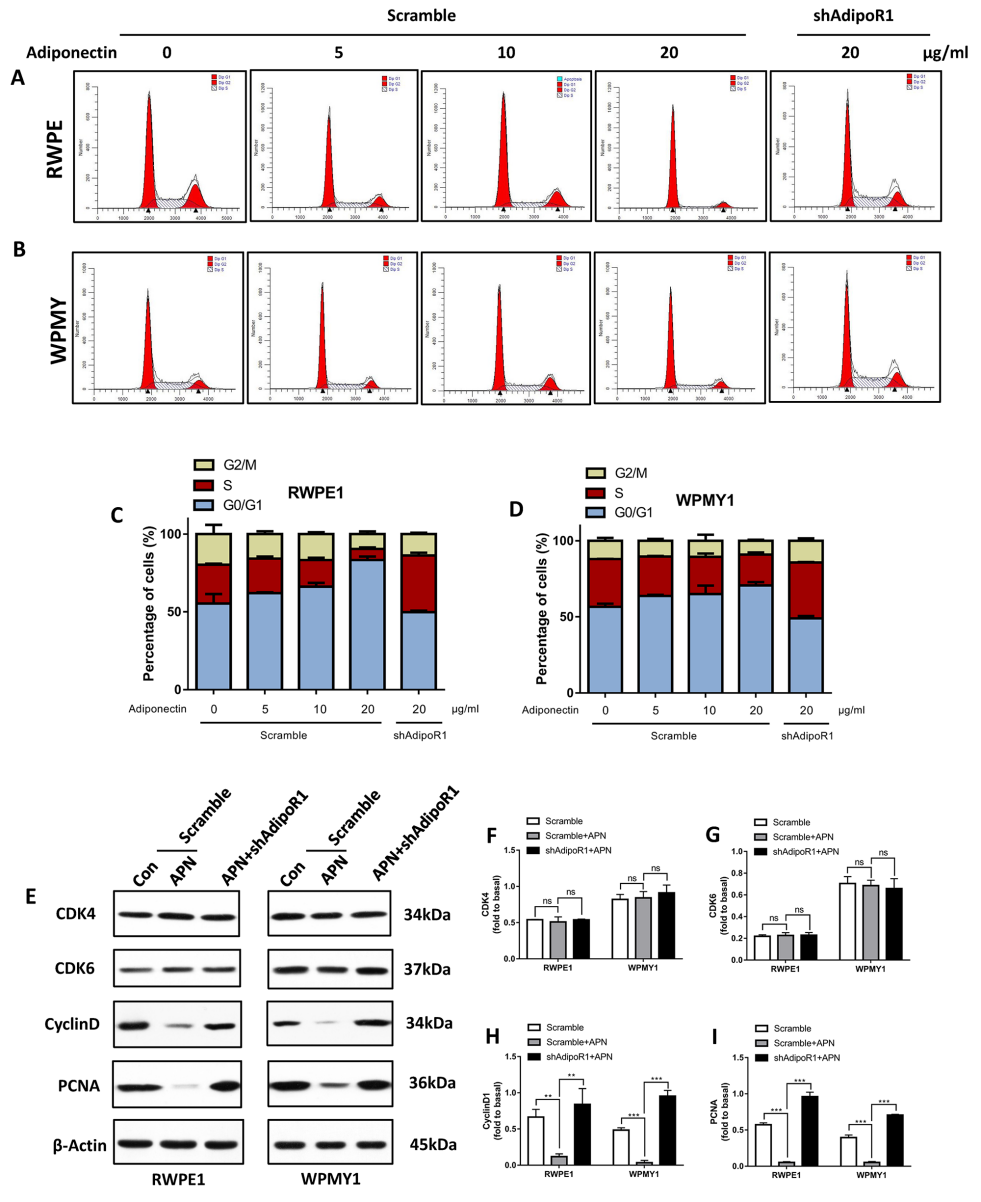
Adiponectin arrests prostatic cells in the G_0_/G_1_ phase (**A, B**) Flow cytometry analysis of the cell cycle of RWPE1 and WPMY1 cells with transfection after the addition of different concentrations of adiponectin. (**C, D**) The cell cycle distribution was further analyzed using ModFit LT software. The results are expressed as the mean±s.d. of three independent experiments. (**E**) Cells were incubated with 10 ng/ml EGF for 1 h followed by adiponectin treatment (APN) or PBS (Con) for 2 h. Then, the cellular extracts were analyzed for expression of CDK4/6, cyclinD1 and PCNA. (**F, G, H, I**) Quantification for expression of CDK4/6, cyclinD1 and PCNA. The results are expressed as the mean±s.d. of three independent experiments (one-way analysis of variance followed by Bonferroni post-tests; **p<0.01, ***p<0.001, ns, not significant).

As indicated in Figure 4, cells were incubated with 10 ng/ml of EGF and treated with different concentrations of adiponectin for 12 h before flow cytometry. Comparing adiponectin-treated cells with control cells, the average proportion of cells in the G_0_/G_1_ phase was increased, and the average proportion of cells in the S phase was decreased with an increase in adiponectin concentrations. Importantly, AdipoR1 knockdown facilitated the EGF-induced G1/S-phase transition.

Next, we examined protein markers of proliferation and the cell cycle by immunoblotting (Figure 4E). Contrary to the effect of EGF, the addition of adiponectin markedly suppressed the expression of cyclinD1 and proliferating cell nuclear antigen (PCNA) (Figure 4F and 4G). However, adiponectin did not impact the expression of cyclin-dependent kinase 4 and 6 (CDK4/6) (Figure 4H and 4I). CyclinD1 is a regulatory protein of the cell cycle that dimerizes with CDK4/6 to regulate the transition from the G1 to S phase, suggesting that cyclinD1 might be one of the targets for adiponectin. These results reveal that adiponectin effectively arrests prostate cells in the G_0_/G_1_ phase and inhibits entry into the S phase. With adiponectin deficiency, the EGF-mediated cell-cycle advancing effect is relatively strengthened, and cells tend to have a greater replicative potential.

### The role of the MEK-ERK-p90RSK axis in mediating adiponectin effects in prostate cells

**Figure 5:**
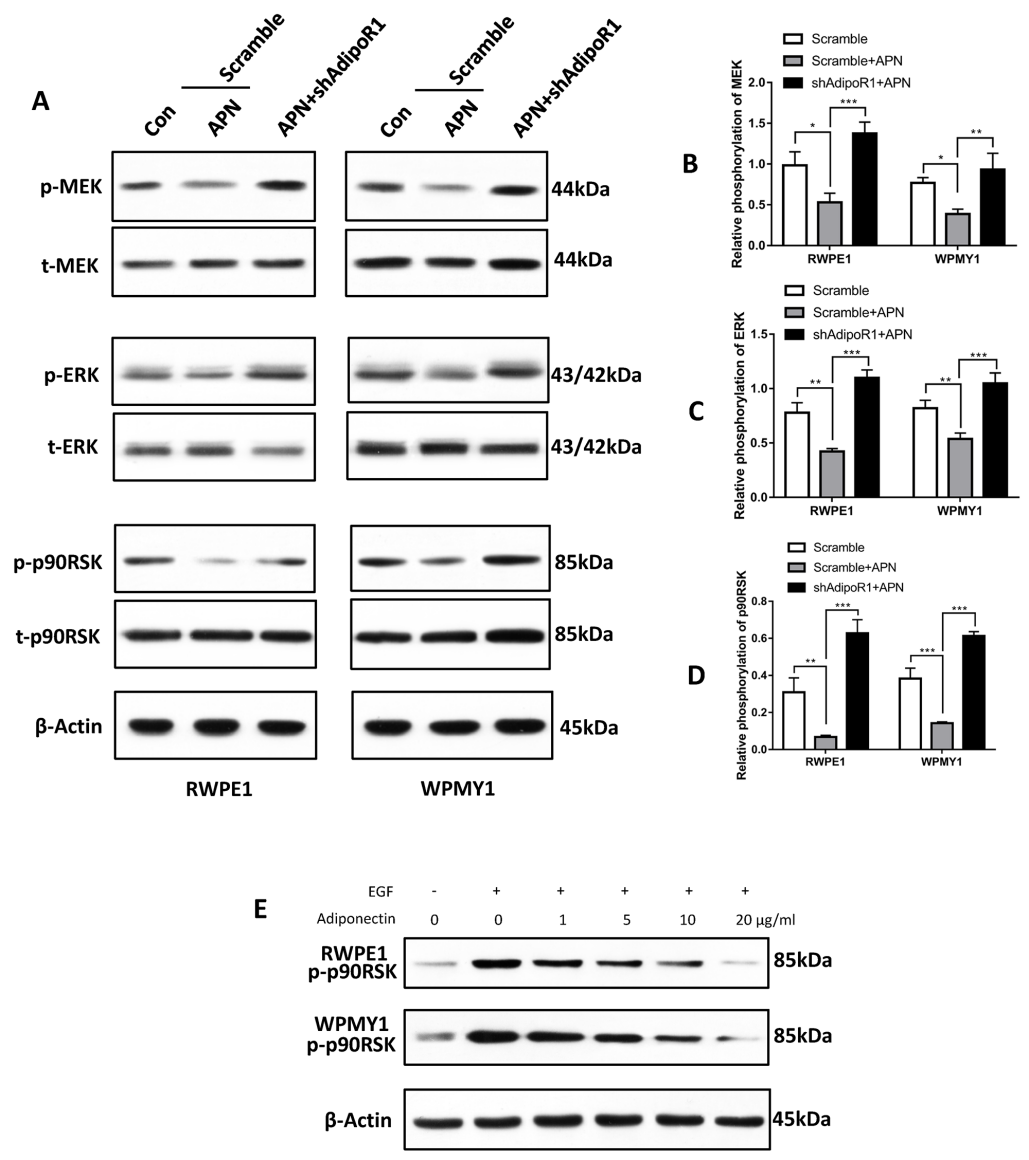
Adiponectin negatively regulates the MEK-ERK-p90RSK axis (**A**) Cells were incubated with 10 ng/ml EGF for 1 h followed by adiponectin (APN) or PBS (Con) treatment for 2 h. Then, the cellular extracts were analyzed for expression of the indicated proteins. (**B, C, D**) Quantification for phosphorylation of MEK, ERK and p90RSK. The results are expressed as the mean±s.d. of three independent experiments (one-way analysis of variance followed by Bonferroni post-tests; *p<0.05, **p<0.01, ***p<0.001). (**E**) Cells were treated at the indicated conditions for 2 h followed by Immunoblotting for phosphor-p90RSK. This experiment was performed twice with similar results.

Previous studies have reported several signaling pathways involved in adiponectin signal transduction. APPL1 was the first identified molecule to directly interact with AdipoR1, which primarily leads to activation of AMPK, PPARα and 38-MAPK via interacting with Rab5 [12–15]. p90RSK is a downstream effector of extracellular signal-regulated kinase (ERK), which is a serine/threonine kinase member of the S6 ribosomal kinase family. It is known to regulate cell proliferation, apoptosis, cell cycle, mRNA translation, tumor invasion and metastasis and other signalling pathways [16, 17]. In agreement with previous studies [18–20], we found that adiponectin inhibited phosphorylation of mitogen-activated protein kinase/extracellular signal-regulated kinases kinase (MEK), ERK and p90RSK in both RWPE1 and WPMY1 cells (Figure 5A-5D). Furthermore, in agreement with our *in vivo* results, we found that the EGF-induced phosphorylation of p90RSK was attenuated significantly by adiponectin (Figure 5E). Knockdown of AdipoR1 facilitated the phosphorylation of the MEK-ERK-p90RSK axis induced by EGF (Figure 5A-5D). These results suggest an involvement of the MEK-ERK-p90RSK-axis downregulation in adiponectin-AdipoR1 effects.

## DISCUSSION

Recent epidemiological studies have closely linked BPH with obesity [3, 4]. Adiponectin has received much attention due to its inverse association with the consequences of obesity [9]. Our results from case-control study indicated that serum adiponectin levels were negatively associated with the risk of BPH. However, potential limitations should be taken into account when interpreting our study. First, TPV, a measure of clinical BPH and a description of BPE [21], was used as a primary criterion for BPH in our study. However, the pathological hyperplasia of the prostate is an evolutionary process, and no exact cutoff value between the normal prostate and prostate hyperplasia has been defined. It is insignificant to consider serum adiponectin as a biomarker of BPH. Second, our study was limited by a small sample size, partially due to the strict inclusion and exclusion criteria we used to reduce confounders. Larger-scale clinical studies are needed to test these clinical correlations. Additionally, our findings suggest that lower serum adiponectin levels are associated with increased prostate volume rather than symptomatic BPH.

**Figure 6:**
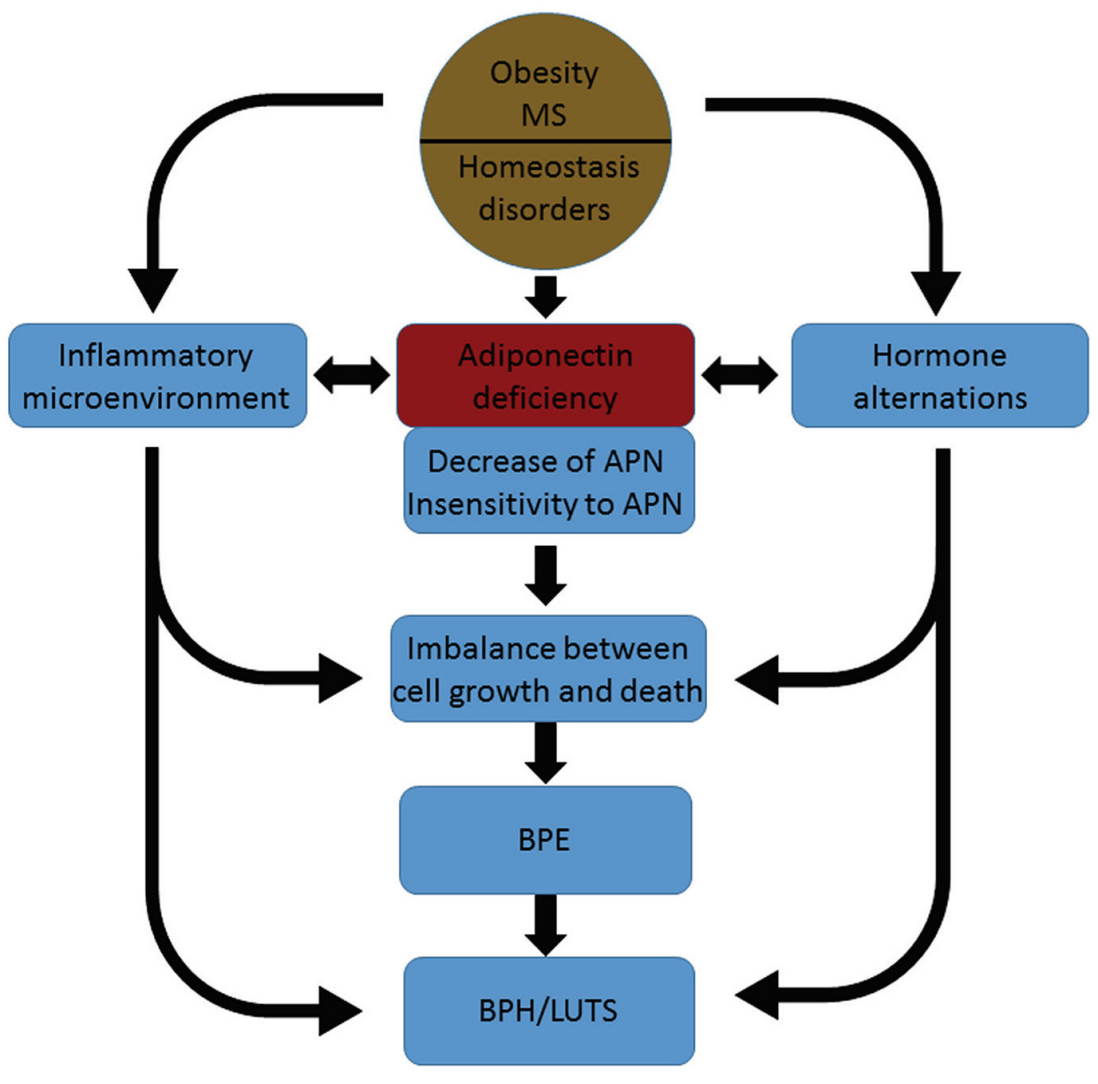
A diagram for our hypothesis that adiponectin deficiency links obesity with BPH APN, adiponectin; MS, metabolic syndrome; BPE, benign prostatic enlargement; BPH, benign prostatic hyperplasia; LUTS, lower urinary tract symptoms.

In agreement with previous studies [7, 22], we found that serum adiponectin levels were negatively associated with BMI levels. Obesity is also an important risk factor for BPH [23]. Higher BMI levels are associated with an increased risk of BPH; lower serum adiponectin levels are associated with increased BMI levels and an increased risk of BPH. These two characteristics may be paralleled and independent with each other. However, there is a possibility, as shown in Figure 6, that adiponectin deficiency might be a bridge between obesity and BPH, linking the two pathological conditions. Further studies are needed to fully elucidate these pathological connections. A number of previous studies have shown that serum adiponectin levels are negatively associated with the risk of prostate cancer and the differentiation grade [24, 25]. Adiponectin may protect the prostate from hyperplastic diseases that have a limited or limitless proliferation capacity.

We found that BPH tissues and normal prostate cell lines had a large extent expression of AdipoR1 than of AdipoR2 in our preliminary experiments (Supplementary Figure 1). Then, the tissue microarray analysis showed a decreased expression of AdipoR1 in hyperplastic tissues compared with normal tissues. Additionally, the expression of p-p90RSK was inversely correlated with the expression of AdipoR1, which is in agreement with the *in vitro* results. AdipoR1 was abundantly expressed on prostate tissues, which suggested that adiponectin may act on prostate cells either directly or indirectly. Indeed, adiponectin has been reported to have antitumor activity via suppressing neovascularization [26, 27] and increasing anti-oxidative defense mechanisms [28]. Regardless of these indirect effects, which are important in the progression of prostate cancer, there is evidence that adiponectin inhibits proliferation of various cell lines in vitro [18, 29, 30] including prostate cancer cells [11, 29]. Adiponectin generally has protective effect in the prostate. AdipoR1 downregulation or serum adiponectin reduction might result in abrogation of these protective effects, leading to the emergence of various diseases. However, the expression of adiponectin receptors may be affected by plasma insulin levels, obesity and other metabolic alternations [30, 31]. We have a shortage that we did not include the effect of metabolic syndrome on AdipoR1 expression. A more thorough assessment of metabolic alternations is needed to eliminate confounders.

As expected, we found that adiponectin directly inhibited the proliferation of normal prostate epithelial and stromal cells, it exerted an opposite effect of EGF. We also found that adiponectin arrested the cell cycle in the G_0_/G_1_ phase. With adiponectin deficiency, the two different types of prostate cells tended to have a greater proliferation ability. Furthermore, we found an inverse association between adiponectin and p-p90RSK both in studies completed *in vitro* and *in vivo*. Adiponectin may inhibit cell proliferation and the cell-cycle progress by inhibiting the MEK-ERK-p90RSK signaling pathway.

In conclusion, we showed a possible signaling correlation exists, involving adiponectin signaling and MEK-ERK-p90RSK signaling, to mediate the anti-proliferation effect of adiponectin in prostate cells. A deficiency in adiponectin results in the facilitation of cell growth and multiplication. The mechanism implied by our experimental results may explain the clinical association among obesity, adiponectin deficiency and BPH. Our results provide a possible explanation for the pathogenesis of BPH. Strategies aimed at correcting adiponectin deficiency with lifestyle modifications or adiponectin receptor agonists seem to be a translational possibility for BPH prevention and therapy.

## MATERIALS AND METHODS

### Antibodies and regents

Antibodies against PCNA (2586), phosphor-MEK1/2 (9154), phosphor-p90RSK (11989) and phosphor-MSK1 (9595) were obtained from Cell Signaling Technology (CST, MA, USA). Antibodies against AdipoR1 (ab126611), p90RSK (ab32114), cyclinD1 (ab134175), CDK4 (ab108357), CDK6 (ab124821), total-ERK1/2 (ab184699), phospho-ERK1/ (ab76299) and total-MEK1/2 (ab178876) were obtained from Abcam (Cambridge, UK). Recombinant human adiponectin (1065-AP) and recombinant human epidermal growth factor (EGF, 236-EG) were obtained from R&D System.

### Patients

Between August 2015 and August 2016, 48 Chinese men with newly diagnosed BPH and 50 control subjects were enrolled in the case-control study at the department of urology, Shanghai Ninth People’s Hospital, Shanghai Jiao Tong University School of Medicine. The diagnosis was based on TPV ≥30 ml and IPSS ≥7 [32]. Fifty control subjects were recruited among men who were admitted to our department at the same time but displayed no evidence of BPH on the basis of TPV <30 ml and IPSS<7. The age of subjects was limited to between 60 and 80 years old. TPV was detected by transrectal ultrasonography and determined using the accurate method [33]. The IPSS is a questionnaire to assess LUST. Men were excluded from the study if they had a history of prostate biopsy, transurethral surgery, diabetes, hypertension, any form of cancer, atherosclerosis, non-alcoholic fatty liver disease, prostatitis, recent urinary infection. Patients with current usage of 5α-reductase inhibitors, anti-androgen drugs or metformin and patients with body temperature ≥37.5 °C, fasting plasma glucose ≥6.1 mmol/L, systolic blood pressure≥140 mmHg or diastolic blood pressure≥90 mmHg, triglycerides≥1.7 mmol/L, HDL≤0.9 mmol/L, CRP≥10 mmol/L or PSA≥10 ng/ml were also excluded.

An in-person interview was conducted after patients were admitted to our hospital using a structured questionnaire including name, age, medical history, pharmacohistory, IPSS, physical activity, smoking and drinking history. Body weight (kg), body height (cm), body temperature (°C) and blood pressure (mmHg) were measured using standard equipment, and the BMI was calculated as the weight divided by height^2^ (kg/m). The BMI was classified according to guidelines for the Asian Pacific population (International Association for the Study of Obesity, World Health Organization; underweight, <18.5; normal, 18.5 to <23; at risk of obesity or overweight, 23 to<25; obese, ≥25) [34]. The IPSS was classified as follow: no symptoms, 0; mild symptoms, 1-7; moderate symptoms, 8-19; severe symptoms, 20-35 [35]. The Q_max_ was measured by uroflowmetry [21]. Blood samples were collected after overnight fasting before operation. Fasting blood glucose, PSA, CRP, HDL, LDL, total cholesterol and triglyceride were measured using the standard measurements of our hospital. For adiponectin measurements, partial blood samples were collected in pro-coagulation tube with gel and centrifuged at 3000 rpm for 5min to obtain serum samples. Samples were stored at -80 °C until all samples were completed. All patients were given written informed consent before examinations. This study was approved by the Ethics Committee of Shanghai Jiao Tong University School of Medicine.

### Enzyme-linked immunosorbent assay

Serum adiponectin levels were measured according to the manufacturers’ instructions, using an enzyme-linked immunosorbent assay kit (RayBiotech, Norcross, GA, USA) specific for humans (sensitivity was 25 pg/ml). Each sample was tested in triplicate. The inter-assay coefficient of variation was less than 10 %, and the intra-assay coefficient of variation was less than 12 %.

### Tissue microarray analysis

Expression of AdipoR1 and phosphorylated p90 ribosomal S6 kinase (p-p90RSK) was determined with tissue microarray analysis obtained from US Biomax (PR807b, containing 10 normal prostatic tissue cores and 21 hyperplasia prostatic tissue cores). IHC staining was performed according to the Diaminobenzidine staining procedure of Dako EnVision detection system (Dako, Denmark). Antigen retrieval was processed by heating method with 10 mM sodium citrate buffer (pH 6.0) for 30 min. Then, primary antibodies of AdipoR1 (1:200) or phospho-p90RSK (1:400) were used. Negative controls were acquired omitting the primary antibody. As previously reported [36, 37], the histochemistry score (H-Score) of each core was calculated with QuantCenter software (version 2.0, 3DHISTECH Ltd., Hungary), which can recognize and analyze the staining intensity (0, negative; 1, weak; 2, moderate; 3, strong) and positive staining area (pixels). H-Score = ∑ (PI×I) = (percentage of cells of weak intensity ×1) + (percentage of cells of moderate intensity ×2) + (percentage of cells of strong intensity ×3). The scoring was performed by an assessor who was blinded to the design and process of experiments. The study was approved by the Ethics Committee of Shanghai Jiao Tong University School of Medicine.

### Cell culture and treatment

The human prostatic epithelial cell line (RWPE1) and human prostatic stromal cell line (WPMY1) were purchased from American Type Culture Collection (ATCC, USA). RWPE1 was grown in prostatic epithelial cell medium (PEpiCM, ScienCell) with 1 % prostatic epithelial cell growth supplement containing various growth factors (PEpiCGS, ScienCell) and 1 % penicillin/streptomycin (ScienCell). WPMY1 cells were grown in high-glucose DMEM (HyClone, CA) supplemented with 10 % FBS (HyClone, CA) and 1 % penicillin/streptomycin. Cells were cultured at 37 °C with 5 % CO_2_ in 100 mm culture dishes.

As previously reported, EGF receptors were expressed on RWPE1 cells, and EGF stimulated the growth of RWPE1 cells by activating the ERK signaling pathway [38]. Thus, in our experiments, RWPE1 cells and WPMY1 cells were cultured in PEpiCM without PEpiCGS and serum-free medium as a negative control, or treated with 10 ng/ml EGF as a positive control that could activate the ERK signaling pathway.

### RNA interference and the generation of AdipoR1-knockdown cells

The lentiviral vectors containing the shRNA of human AdipoR1 (Lenti-shAdipoR1, sequence 5’-TGGCTCTTTCACACCGTCT-3’) or a scrambled sequence (Lenti-Control) were constructed by Asia-Vector Biotechnology (Shanghai, China). The lentiviral vectors were harvested every 48 h and 72 h after packaging into HEK293T cells. RWPE1 and WPMY1 cells were plated in 6-well plates at a density of 5×10^5^ cells per well and cultured for 24 h. The next day, cells were infected with Lenti-shAdipoR1 or Lenti-Control with 8μg/ml polybrene (Santa Cruz, CA) for 24 h at a multiplicity of infection (MOI) of 10:1. Then, the infected cells were selected with 5 μg/ml puromycin (InvivoGen, USA) for 6 days to obtain stable knockdown cell lines (RWPE-shAdipoR1, RWPE-Scramble, WPMY-shAdipoR1 and WPMY-Scramble). Transfection efficiency was evaluated under a fluorescence microscope. The knockdown efficiency was confirmed by western blotting (Figure 3A and 3B).

### Cell proliferation assay

Cells were plated in 96-well plates at 2000 cells per well in 100 μl medium with 10 ng/ml EGF, and treated with different concentrations of adiponectin. Cell proliferation was analyzed by the Cell Counting Kit-8 assay according to the manufacturer’s instructions (C0037, Beyotime Biotechnology, China). Briefly, 10 μl of CCK-8 solution was added to each well and cells were cultured at 37 °C for 1 h. The absorbance at 450 nm was measured with a Varioskan Flash Spectral Scanning Multimode Reader (Thermo).

### Cell cycle analysis

Cells were plated in 60 mm culture dishes at a density of 1×10^6^ cells per dish. After starvation for 12 h and treatment with the indicated conditions for 12 h, the treated cells were collected and washed twice with cold PBS. Then, a single cell suspension was fixed with 70 % cold ethanol for 12 h. Finally, according to the manufacturer’s instructions (C1052, Beyotime Biotechnology, China), the cells were stained with a propidium iodide (PI) mixture for 30 min at 37 °C before flow cytometry analysis (Beckman Coulter Flow Cytometer, Krefeld, Germany). The cell cycle distribution was further analyzed with ModFit LT (V4 1.7, Verity Software House, Topsham, ME).

### Protein isolation and immunoblotting

Cells were collected and lysed on ice with RIPA Lysis and Extraction buffer (Thermo Scientific) and with Halt Protease and Phosphatase inhibitor Cocktail (Thermo Scientific). After centrifugation for 10 min at 10000 g and 4 °C, the supernatants were collected, and the total protein concentration was determined with a BCA protein assay kit according to the manufacturer’s instructions (P0010S, Beyotime Biotechnology, China). The lysates were loaded and separated by SDS–PAGE gel electrophoresis, followed by transfer to PVDF membranes (Millipore). Membranes were blocked with TBS/T buffer containing 5% milk and incubated with primary antibodies against AdipoR1 (1:1000), PCNA (1:2000), cyclinD1 (1:1000), CDK4(1:1000), CDK6(1:1000), phosphor-MEK1/2 (1:1000), total-MEK1/2 (1:1000), phosphor-ERK1/2 (1:1000), total-ERK1/2 (1:1000), phosphor-p90RSK1/2 (1:1000), total-p90RSK1/2 (1:1000), tublin (1:2000) and β-actin (1:2000) overnight at 4 °C. Then, membranes were washed and incubated with horseradish peroxidase-conjugated secondary antibody (1:2000, Jackson) at room temperature for 2 h and finally detected by a chemiluminescent method with ECL Western Blotting Substrate (Thermo Scientific). The blots were visualized and analyzed using GelPro Imaging System (CBIO, Beijing, China) and GelPro 1D software version 4.5.

### Statistical analysis

Data were expressed as mean values with standard deviation or numbers with proportions. Comparisons were conducted using Student’s t-test or a nonparametric test (Mann-Whitney U test or Kruskal-Wallis test) for continuous variables and χ^2^ tests for categorical variables. Analysis of variance (ANOVA) followed by a Dunnett post-test was performed for multiple comparisons. Two-tailed continuous bivariate correlations were determined by the Pearson’s correlation coefficient. To explore the predictors of prostate volume, we used multivariate linear regression, which was determined by the stepwise selection of significant predictors in univariate analysis. The non-normally distributed variables were log-transformed. We also conducted univariate and multivariate logistic regression using morbid status as the outcome variable and adiponectin as well as other possible confounders as the predictor variables. A two-side p value less than 0.05 was considered statistically significant. All analyses were performed using SPSS, version 21.0 (SPSS Inc, Chicago, Illinois, USA).

## SUPPLEMENTARY MATERIALS FIGURE


