# Supplementary material for: Lack of adiponectin and adiponectin receptor 1 contributes to benign prostatic hyperplasia
Source: Oncotarget. 2017 Aug 3;8(51):88537–51. doi: 10.18632/oncotarget.19877 (PMC5687625; doi:10.18632/oncotarget.19877)
Supplement: Supplementary file 1 [file oncotarget-08-88537-s001.pdf]

Lack of adiponectin and adiponectin receptor 1 contributes to benign prostatic hyperplasia

SUPPLEMENTARY MATERIALS

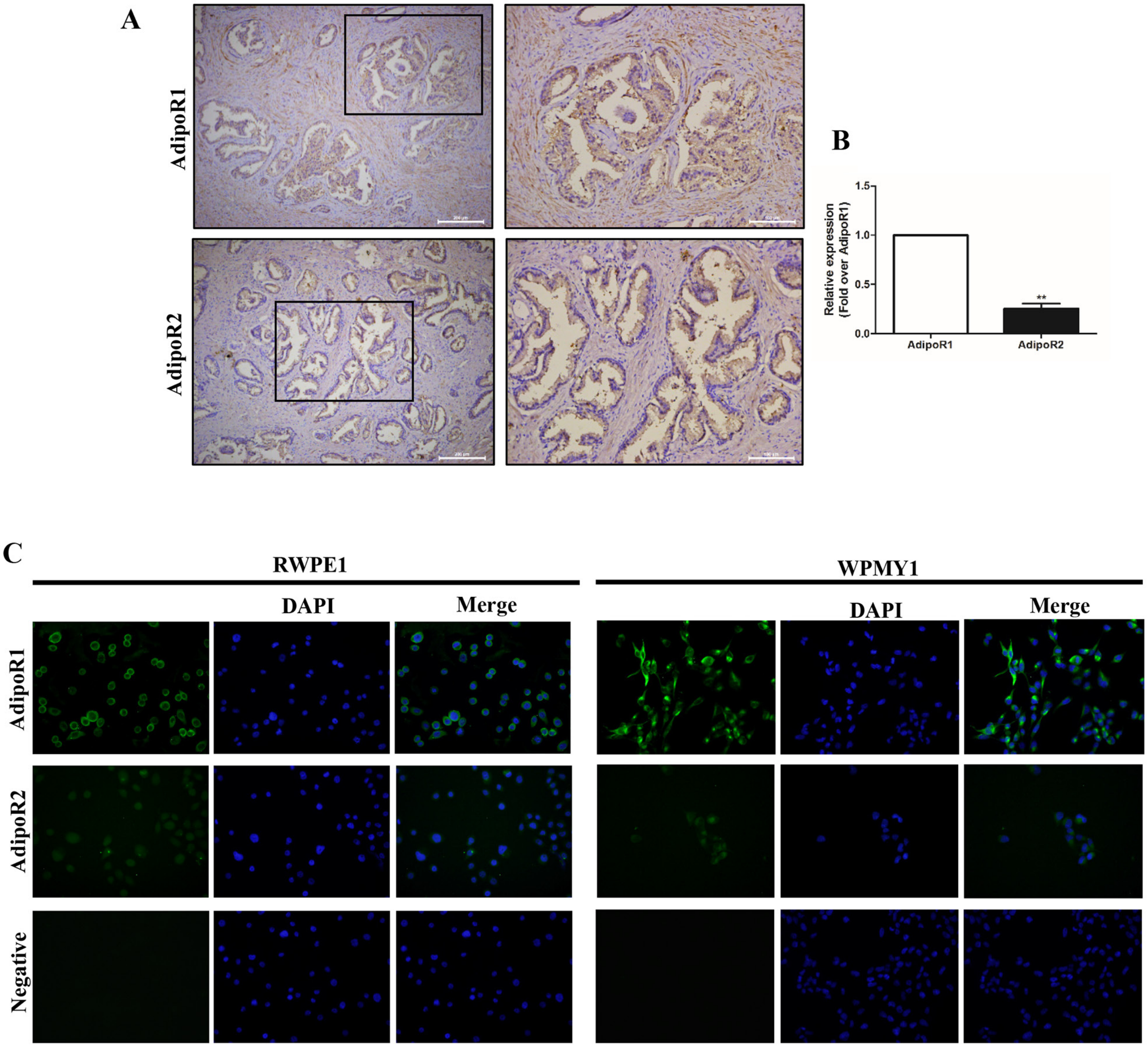

Supplementary Figure 1: Expression of adiponectin receptors in prostatic tissues and cells. **(A)** IHC staining for AdipoR1 and AdipoR2 on human postoperative BPH tissue samples. Scale bars, 100 μm or 200 μm. **(B)** Semi-quantitation based on the average of optical density (AOD) measured by Image J software (n=10, Student's t-test, **p<0.01). **(C)** Immunofluorescence staining (green) for AdipoR1 and AdipoR2 in RWPE1 and WPMY1 cells, DAPI staining (blue) for nucleus. Magnification, ×400.
